# Spontaneous remission of choroidal involvement by chronic myelomonocytic leukemia: a case report

**DOI:** 10.3389/fonc.2024.1399894

**Published:** 2024-05-02

**Authors:** Elisa Diral, Gloria Catalano, Maria Vittoria Cicinelli, Andrea Distefano, Sara Mastaglio, Luca Vago, Maria Teresa Lupo Stanghellini, Massimo Bernardi, Maurilio Ponzoni, Fabio Ciceri, Matteo G. Carrabba

**Affiliations:** ^1^ Department of Hematology and Bone Marrow Transplantation, IRCCS San Raffaele Scientific Institute, Milan, Italy; ^2^ Vita-Salute San Raffaele University, Milan, Italy; ^3^ Department of Ophthalmology, IRCCS San Raffaele Scientific Institute, Milan, Italy; ^4^ Pathology Unit, IRCCS San Raffaele Scientific Institute, Milan, Italy

**Keywords:** chronic myelomonocytic leukemia, ocular involvement, personalized medicine, multimodal imaging analysis, watch and wait approach

## Abstract

Chronic myelomonocytic leukemia (CMML) is a rare hematological disorder characterized by variable risk of evolution to acute myeloid leukemia; to date, allogeneic stem cell transplantation is the only curative treatment. We report a case of choroidal involvement in a woman affected by CMML and presenting only with visual impairment. The patient was initially evaluated for an intensive therapeutic approach, but after biopsy the ocular lesion spontaneously regressed. Thus a “watch and wait” strategy was preferred. One year and a half after initial diagnosis, the patient is alive, with stable hematological disease and without any ocular involvement. Therefore, a close, not invasive follow up could be useful to tailor treatment for patients affected by single ocular lesions in CMML.

## Introduction

Chronic myelomonocytic leukemia (CMML) is a rare hematological disorder characterized by variable risk of evolution to acute myeloid leukemia (AML). Among prognostic models, the CPSS (1) and CPSS molecular (CPSS-mol) (2) are the most frequently used in CMML, in order to predict outcome and to guide treatment. Lower risk patients can undergo vigilant observation in the absence of significant cytopenias, while the only curative treatment for high risk patients is represented so far by allogeneic stem cell transplantation (allo-SCT) (3, 4). Extramedullary localization in CMML is uncommon and organs such as spleen, liver, skin, and lymph nodes are usually involved (5–8). Ocular involvement in CMML is rare and is associated with poor prognosis and increased risk of evolution to AML (9, 10). Here we report the case of a patient with single ocular involvement by CMML who did not receive specific treatment due to spontaneous remission of the lesion.

## Case report

**Figure 1:**
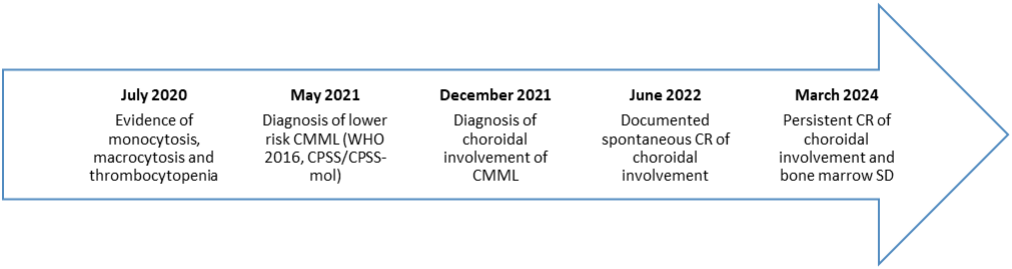
Timeline of relevant clinical data. CMML, chronic myelomonocytic leukemia; CPSS, CMML-specific prognostic scoring system; CPSS-mol, CMML-specific prognostic scoring system – molecular; CR, complete remission; SD, stable disease.

**Figure 2:**
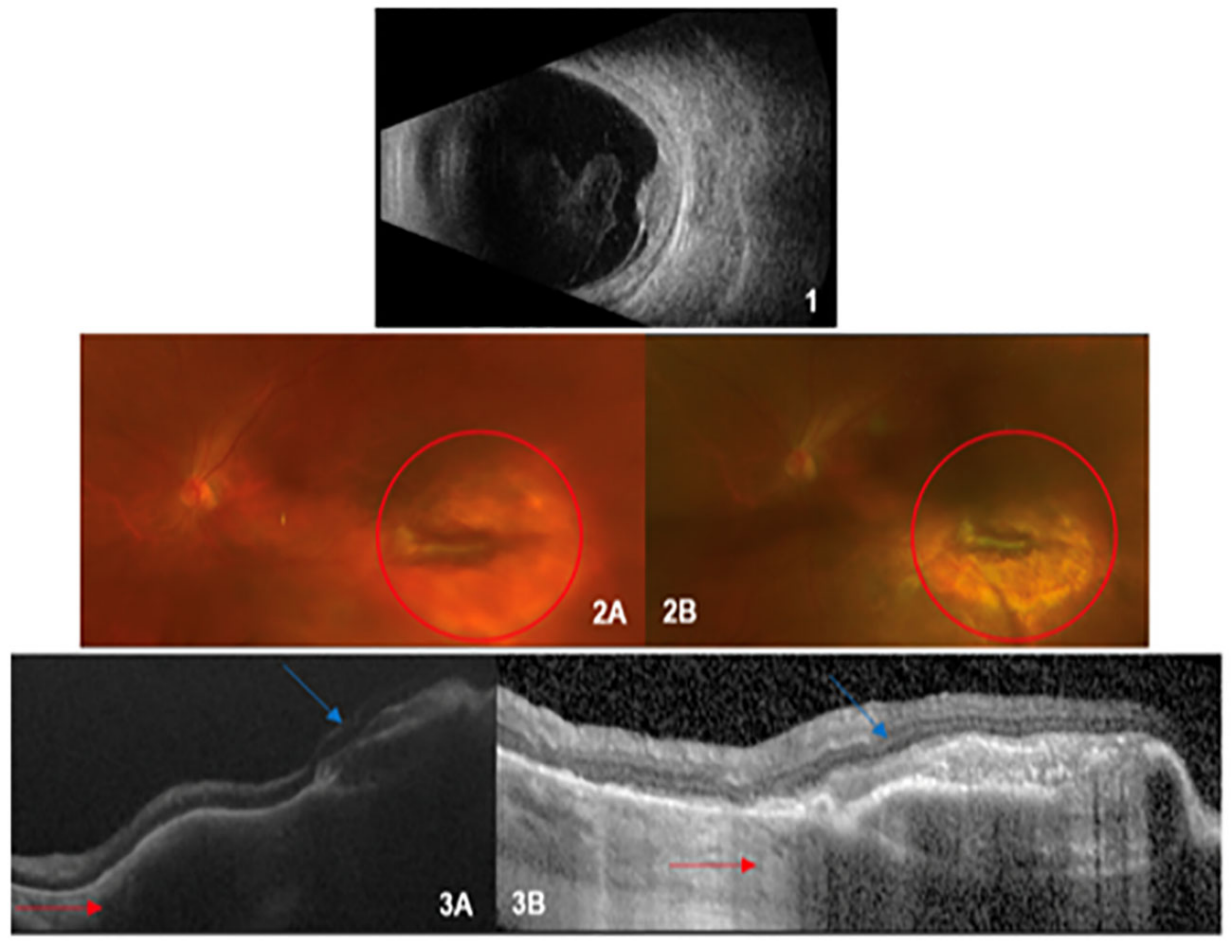
1) *Scan Echography*: raised lesion in the retinal plane with medium-low internal reflectivity with inferior exudate retinal detachment and mild vitritis. 2) *Ultra-wide field retinography* (baseline and follow-up): on the left (2A), a scarring lesion surrounded by a shaded retinal area (red circle); on the right (2B), disappearance of the shaded area around the permanent scar. 3) *Optical Coherence Tomography (baseline and follow up)*: voluminous hyporeflective area under the retinal layer in the left image (3A), dramatically reduced after follow up in the right one (red arrow, 3B); the scarring lesion has remained stable (blue arrow).

A 65-year-old woman was referred to our hematology service in July 2020 after a severe COVID-19 infection requiring intensive care (**Figure 1**). The patient presented with mild monocytosis and thrombocytopenia. Her complete blood counts (CBC) were: PLT 60 x 10^3^/µl, WBC 6 x 10^3^/µl, ANC 3.6 x 10^3^/µl, ALC 1.2 x 10^3^/µl, Monocytes 1.8 x 10^3^/µl, Hb 12 g/dl, and MCV 100.5 fL. Her physical examination was negative. After a ten month follow up showing persistent monocytosis, thrombocytopenia and macrocytosis, secondary causes were ruled out (autoimmune or infectious diseases), and a bone marrow evaluation was diagnostic for CMML-0 dysplastic type according to WHO 2016 classification in May 2021. Cytogenetics and FISH were normal, while molecular testing showed oncogenic mutations in TET2 and SRSF2 genes. CPSS and CPSS-mol risk score were low, so a watch and wait strategy was established. Soon after, the patient complained of visual loss in her left eye; the dilated fundus evaluation showed mild vitritis and a hypo-pigmented choroidal lesion with exudative retinal detachment in the mid-temporal periphery. Multimodal imaging including A- and B-scan ultrasound, dye angiography and optical coherence tomography suggested an infiltrative lesion (**Figure 2** – panels 1, 2A, 3A). A retino-choroidal biopsy, performed in December 2021, disclosed substantial involvement by medium-sized mononuclear cells, immunoreactive for CD45, CD14, and CD68R. The absence of CD34 and CD117 immunoreactivity ruled out a myeloid sarcoma and the ophthalmic lesion resulted consistent with involvement by CMML. The patient was evaluated for intensive chemotherapy and subsequent consolidation with allo-SCT considering the poor prognosis associated with ocular involvement (9, 10). However, one month later (January 2022), a new ophthalmic evaluation revealed a spontaneous decrease in the lesion size. In addition, a reduction in visual acuity from 20/28 to 20/100 was reported, due to damage to the outer retinal layers resulting from subretinal exudation. CBC and marrow disease were stable and, considering the low risk according to CPSS-mol, we opted for a “watch and wait” strategy. The CMML ophthalmic lesion completely disappeared in June 2022 (**Figure 2** – panels 2B and 3B). At last follow up in March 2024 the patient had stable CBC; a new bone marrow evaluation, performed in November 2023, confirmed stable chronic disease, without signs of leukemic evolution and the left choroidal lesion was in complete remission with a good vision recovery.

## Discussion

Extramedullary localization in CMML is uncommon and its prognostic value is unclear; a retrospective study by Mayo clinic demonstrated that non hepatosplenic extramedullary disease in patients affected by CMML did not impact on both overall and leukemia free survival (11). Ophthalmic localizations are rare but associated with poor prognosis (9, 10) and can lead to aggressive therapeutic approaches, particularly in young patients. In our case, the spontaneous remission of the single ocular lesion together with a favorable molecular risk score, prompted us to opt for a “watch and wait” approach. This resulted in preservation of a good health related quality of life, without signs of disease progression at last follow up. In conclusion, our case report describes a spontaneous remission of choroidal involvement by CMML, suggesting that a close, not invasive follow up could be useful to tailor treatment for patients affected by single ocular lesions in CMML.

## Data availability statement

The raw data supporting the conclusions of this article will be made available by the authors, without undue reservation.

## Ethics statement

Written informed consent was obtained from the individual(s) for the publication of any potentially identifiable images or data included in this article.

## Author contributions

ED: Conceptualization, Methodology, Writing – original draft, Writing – review & editing, Investigation, Validation. GC: Writing – original draft. MC: Investigation, Validation, Writing – original draft, Writing – review & editing, Conceptualization. AD: Writing – original draft. SM: Writing – review & editing. LV: Writing – review & editing. ML: Writing – review & editing. MB: Writing – review & editing. MP: Writing – original draft, Writing – review & editing. FC: Supervision, Validation, Writing – review & editing. MC: Methodology, Supervision, Validation, Writing – original draft, Writing – review & editing.
